# Distance and utilisation of out-of-hours services in a Norwegian urban/rural district: an ecological study

**DOI:** 10.1186/1472-6963-13-222

**Published:** 2013-06-17

**Authors:** Guttorm Raknes, Elisabeth Holm Hansen, Steinar Hunskaar

**Affiliations:** 1 National Centre for Emergency Primary Health Care, Uni Health, Uni Research, Kalfarveien 31, 5018 Bergen, Norway; 2 Haraldsplass Deaconess University College, Ulriksdal 10, 5009 Bergen, Norway; 3 Department of Global Public Health and Primary Care, University of Bergen, Box 7800, 5020 Bergen, Norway

## Abstract

**Background:**

Long travel distances limit the utilisation of health services. We wanted to examine the relationship between the utilisation of a Norwegian out-of-hours service and the distance from the municipality population centroid to the associated casualty clinic.

**Methods:**

All first contacts from ten municipalities in Arendal out-of-hours district were registered from 2007 through 2011. The main outcomes were contact and consultation rates for each municipality for each year. The associations between main outcomes and distance from the population centroid of the participating municipalities to the casualty clinic and were examined by linear regression. Demographic and socioeconomic factors were included in multivariate linear regression. Secondary endpoints include association between distance and rates of different first actions taken and priority grades assessed by triage nurses. Age and gender specific subgroup analyses were performed.

**Results:**

141 342 contacts were included in the analyses. Increasing distance was associated with marked lower rates of all contact types except telephone consultations by doctor. Moving 43 kilometres away from the casualty clinic led to a 50 per cent drop in the rate of face-to-face consultations with a doctor. Availability of primary care doctors and education level contributed to a limited extent to the variance in consultation rate. The rates of all priority grades decreased significantly with increasing distance. The rate of acute events was reduced by 22 per cent when moving 50 kilometres away. The proportion of patients above 66 years increased with increasing distance, while the proportion of 13- to 19 year olds decreased. The proportion of female patients decreased with increasing distance.

**Conclusions:**

The results confirm that increasing distance is associated with lower utilisation of out-of-hours services, even for the most acute cases. Extremely long distances might compromise patient safety. This must be taken into consideration when organising future out-of-hours districts.

## Background

In Norway all 429 municipalities are responsible for ensuring an out-of-hours emergency health care service at all times. In the past 20 years more and more neighbouring municipalities have established common casualty clinics to lessen the burden for local doctors, to reduce costs and to improve the quality of out-of-hours services. For many patients this has resulted in longer travel distances to the nearest casualty clinic in evenings, at night and at weekends. Some have a travel distance of more than 100 kilometres. On the other hand, people living in immediate proximity to the casualty clinics have gained access to new well-equipped and well-staffed emergency services.

There is firm evidence that increasing distance is inversely correlated to the use of all kinds of health services, examples include psychiatric care [1], how doctors utilise hospitals [2], and attendance to mammography screening programmes [3]. Similar associations between distance and consultation rate in general practice have been observed [4].

We wanted to examine the effect of distance on utilisation of out-of-hours services in Norway, also when taking demographic and socioeconomic factors into consideration. Our hypothesis was that increasing distance to the casualty clinic is associated with fewer contacts and reduced consultation rates. A quantification of these associations might be important for organisation of primary emergency health care, for example what is the appropriate maximum size of an area covered by a casualty clinic. This knowledge might also be taken into account when dividing the costs between participating municipalities.

## Methods

We performed an ecological study in order to examine the correlation between distance and the utilisation of a casualty clinic. In Norway, casualty clinics (“legevakt”) are emergency primary care centres that handle all kinds of medical out-of-hours inquiries, including life-threatening incidents [5]. Data were collected from the “Watchtower project”, a database of a sentinel network for monitoring emergency primary health care activity in Norway. The database was established by the National Centre for Emergency Primary Health Care in 2007. The Watchtowers are seven out-of-hours districts that have been selected to be representative for the entire primary health care emergency service in Norway. The development and implementation of this database have been described in detail elsewhere [5]. The Watchtower project was approved by the Regional Ethics Committee for Medical Research and by the Norwegian Social Science Data Services.

**Figure 1:**
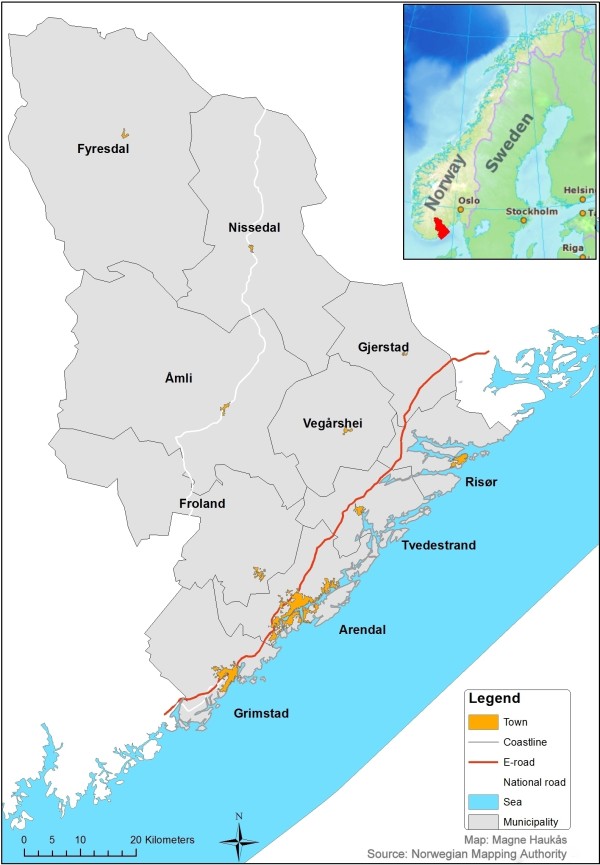
Map of the municipalities covered by Arendal out-of-hours district.

In this study data from the casualty clinic in Arendal were extracted from the Watchtower database, from January 2007 through December 2011. This clinic is located in conjunction with Sørlandet Hospital Arendal and serves ten municipalities in southern Norway (see map) (Figure 1), covering a total population of 90 023 (January 1st 2011). For every single first contact, trained staff (registered nurse or other) registered age and gender of the patient, time of contact, priority degree given, and first action taken. A computer program exclusively designed for this purpose was used. Excel files containing the data were sent by email once per month to the National Centre for Emergency Primary Health Care, where the data were checked for errors before they were added to the database. Procedures for collecting delayed or missing data were established to improve the completeness database.

Home addresses of patients were not registered more accurately than the resident municipality. The road distance between the population centroid for each municipality and Arendal casualty clinic was calculated by Statistics Norway with methods applying ArcView, a geographic information system (GIS) [6]. Although less reliable in rural areas, this method is shown to be superior to geometric centroid and population polygon methods when the exact address is unknown [7]. Main outcomes were also tested for alternative distance measures. These were travel time from the population centroid calculated by Statistics Norway and the road distance in kilometres from the town hall of each municipality to Arendal casualty clinic calculated using Google maps.

The main outcomes were frequencies of all first contacts with the communication centre of the out-of-hours district and frequencies of face-to-face doctor consultations in each of the ten municipalities comprising Arendal out-of-hours district. The rates were calculated by dividing aggregated total number of contacts and consultations for each municipality in each year from 2007 to 2011 by population January 1st the same year. Secondary outcomes include rates of different first actions taken other than face-to-face consultation and rates of the different priority grades. Other first actions taken were telephone consultation with doctor, doctor assisted ambulance, doctor’s home visit, nurse consultation, or telephone consultation with nurse. The degree of urgency was set according to the Norwegian Index for Medical Emergency Assistance [8]. Each call to, or contact with a Watchtower is classified by priority degree with colour codes “Red”, “Yellow”, or “Green”. Red is defined as an “acute” response, with the highest priority. Yellow colour is defined as an "urgent" response, with a high, but lower priority. Green colour is defined as a “non-urgent” response.

Data on potential demographic and socioeconomic confounders for each municipality were obtained from official statistics (Statistics Norway [9] and The Norwegian Institute of Public Health [10]). We considered the following available potentially confounding factors as mutually independent and relevant for multiple linear regression on the main outcomes: Proportion of population more than 80 years old, number of general practitioners per 10 000 inhabitants, proportion of population with elementary school as highest completed education, median gross income and income inequality expressed by the Gini coefficient [11].

Subgroup analyses for rate of all contacts were performed for age and gender. The frequency of contacts in each age group was based on data on age distribution from the 2011 census. For practical reasons we used the same age stratification as Statistics Norway.

We were able to obtain data for all years on number of primary care doctor full-time equivalents (FTEs) per 10 000 inhabitants. Data for 2011 were not available for median gross income. Last observation carried forwards (figures from 2010) were used in multivariate regression for this variable. Data from the 2011 census was applied for all years in multivariate analysis for the percentage of population above 80 years old and the proportion of people who had no more education than compulsory elementary school. The Gini coefficient for each municipality for 2009 was used on all years.

### Statistical methods

PASW Statistics 18 and Excel 2010 software were used to analyse data. All outcomes that form basis for linear regression are number of events per year, which are assumed to be Poisson distributed. The rates of our main outcomes were so high that a normal distribution will fit the data well. Pearson’s product–moment correlation coefficient, r, and linear regression were used to assess any association between distance from municipality to out-of-hours-centre and contact rates for the years 2007–2011 for each municipality. A natural logarithm transformation was used for curve estimation when correlation analysis resulted in a higher Pearson r than for non-transformed data. Regression equations of unadjusted main outcomes were calculated with 95% confidence intervals. For subgroup analyses on age, rate ratios for each age group to all patients were calculated. The alpha level was set to 0.05 (twotailed).

It is known that the frequency of missing data in the Watchtower is generally low (<1%) [12]. We knew that data from seven days in January 2007 and eight days in September 2008 were missing for technical reasons. To compensate for this, all registrations in January 2007 were multiplied with 1.29 and all registrations in September 2008 were multiplied with 1.36. As a sensitivity analysis, all statistical assessments were also performed on non-weighted data. The frequencies of missing data for all variables were registered.

## Results

154 633 Watchtower contacts from Arendal were registered from 2007 through 2011. 1 032 contacts were excluded because information about municipality was lacking. 14 388 contacts were excluded because the patient was a non-Norwegian citizen or was living in a municipality outside Arendal out-of-hours district. 2 347 cases from January 2007 and 2 329 cases from September 2008 were weighted as described in the methods section. 140 245 contacts were eligible for analysis. After weighting, 141 342 contacts were included in the analyses.

Baseline demographic and socioeconomic data are given in Additional file 1.

**Figure 2:**
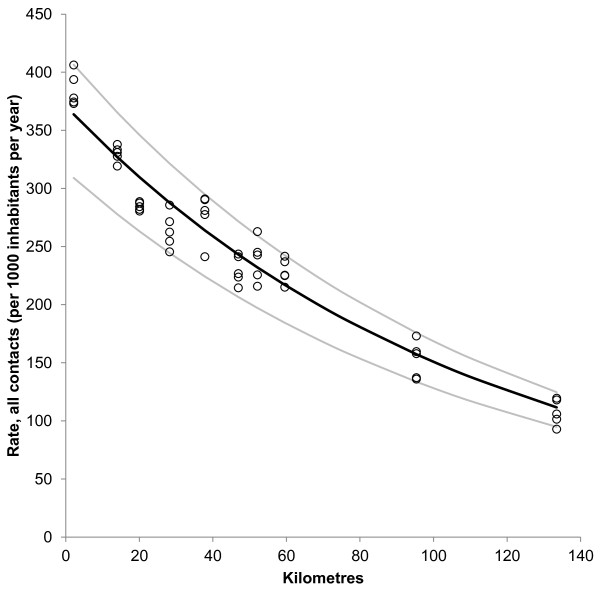
**Distance and rate of all contacts with out**-**of**-**hours service.** Relationship between distance (kilometres) from population centroid of municipality and casualty clinic and rate of all contacts with Arendal out-of-hours service (per 1000 inhabitants per year). Each point represents rate in one municipality for one year. Regression curve (black line) for ln transformed data with 95% confidence interval (grey lines). Ln(contact rate) = 5,92 – 0,009*distance (km). N = 50, R^2^ = 0.95, p < 0.001.

**Figure 3:**
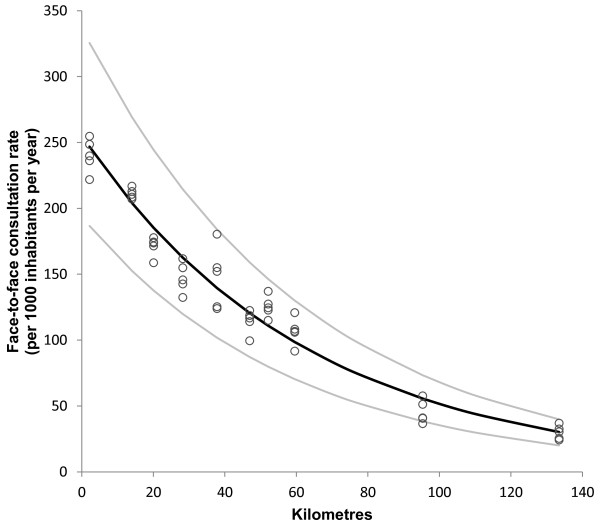
**Distance and face**-**to**-**face consultation rate at the casualty clinic.** Relationship between distance (kilometres) from population centroid of municipality to casualty clinic and face-to-face doctor consultation rate at Arendal casualty clinic (per 1 000 inhabitants per year). Each point represents rate in one municipality for one year. Regression curve (black line) for ln transformed data with 95% confidence interval (grey lines). Ln(consultation rate) = 5,54 – 0,016*distance (km). N = 50, R^2^ = 0.96, p < 0.001.

Figure 2 shows a strong and statistically significant correlation between distance from population centroid of municipality to Arendal casualty clinic and rate of all contacts (R^2^ = 0.95, p < 0.001). The regression equation (unadjusted) shows that moving 77 kilometres away from the casualty clinic reduces the contact rate by 50%. The effect was even greater for doctor consultation rate (Figure 3) (R^2^ = 0.96, p < 0.001). Here, a distance of 43 kilometres results in 50% drop in consultation rate. The most remote municipality (Fyresdal) had a mean contact rate of 28% and a consultation rate of only 12% compared to Arendal municipality, where the casualty clinic is located.

Multiple linear regression analyses for main outcomes are summarised in Tables 1 and 2. No other factor than distance contributed significantly to the variance for total contact rate. Availability of primary care doctors and education level significantly influenced the face-to-face consultation rate. The contribution to variance as expressed by standardised β of both of these was only a tenth compared with the distance from population centroid. The coefficients of determination (R^2^) of the multiple linear regression models were negligibly different from the unadjusted R^2^s.

**Table 1:** Multiple linear regression on rate of all contacts by distance

	**Coefficient (β)**	**95% CI for β**	**Standardised β**	**p**
**Constant**	5.571			
**Distance** (**km**)	-0.010	-0.011 to -0.009	-1.052	< 0.001
**Age** > **80 years** (%)	0.032	-0.001 to 0.065	0.086	0.059
**Income inequality** (**Gini**)	-0.749	-1.017 to 0.351	-0.046	0.32
**Median gross income**	0.098	-0.004 to 0.007	0.044	0.19
**Primary school only**	0.003	-0.004 to 0.011	0.032	0.40
**Primary care doctors per 10 000**	0.001	-0.004 to 0.007	0.021	0.61

Multiple linear regression with rates as the dependent variable. All contacts with Arendal out-of-hours service (ln transformed). **R**^**2**^ = 0.96 for model, p < 0.001.

**Table 2:** Multiple regression on consultation rate by distance

	**Coefficient (β)**	**95% CI for β**	**Standardised β**	**p**
**Constant**	5.083			
**Distance** (**km**)	-0.016	-0.017 to -0.014	-0.928	< 0.001
**Primary care doctors per 10 000**	-0.011	-0.020 to -0.002	-0.092	0.020
**Primary school only** (%)	0.016	-0.003 to 0.028	0.090	0.014
**Age** > **80 years** (%)	0.011	-0.043 to 0.066	0.017	0.68
**Income inequality** (**Gini**)	-0.081	-2.547 to 2.386	-0.003	0.95
**Median gross income**	0.009	-0.239 to 0.257	0.002	0.94

Multiple linear regression with rates as the dependent variable. Face-to-face-consultation with doctor at the casualty clinic (ln transformed). **R**^**2**^ = 0.97 for model, p < 0.001.

**Figure 4:**
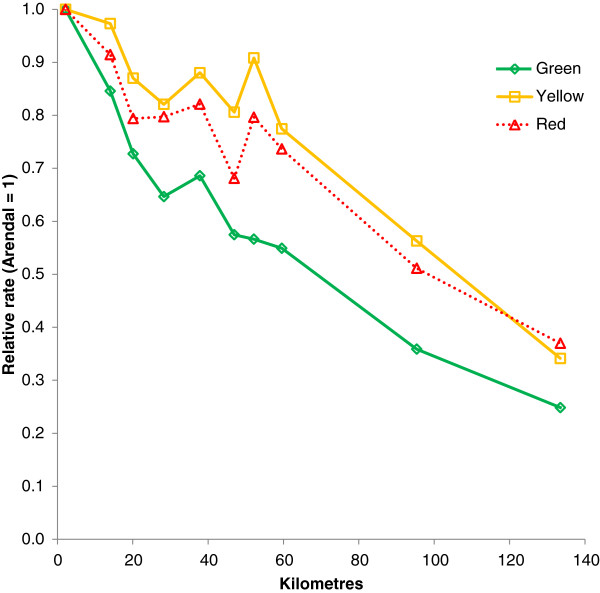
**Distance and priority grades.** Standardised rates of priority grades (mean 2007–2011) by distance from casualty clinic. Arendal municipality is reference (1.0).

Increasing distance from population centroid of the municipality was significantly associated with reduced contact rates for all priority grades, as summarised in Table 3. Figure 4 displays the relationship between distance and rates of the different priority grades relative to the municipality were the casualty clinic is located. The association was most pronounced for green responses, but also yellow and red responses became less frequent with increasing distance. Notably, the R^2^ for red responses was only a fifth of the R^2^s for the main outcomes, indicating that distance to casualty clinic only partially explained the variance in rate of the most acute cases.

**Table 3:** Distance and priority grades

	**Change 0 to 50 km (%)**	**Constant**	**Coefficient (β)**	**95% CI for β**	**R**^**2**^	**p**
**All contacts**	-36.2	5,92	-0.009	-0.010 to -0.009	0.95	<0.001
**Not urgent** (**green**)	-39.3	5.64	-0.01	-0.011 to -0.009	0.91	<0.001
**Urgent** (**yellow**)	-29.5	4.34	-0.007	-0.009 to -0.005	0.48	<0.001
**Acute** (**red**)	-22.1	2.13	-0,005	-0.007 to -0.002	0.19	0.001

Relative change (%) in contact rates of different priority grades when moving 50 kilometres away from the casualty clinic. Simple linear regression for the association between distance and the different priority grades performed on ln-transformed data. N = 50.

**Figure 5:**
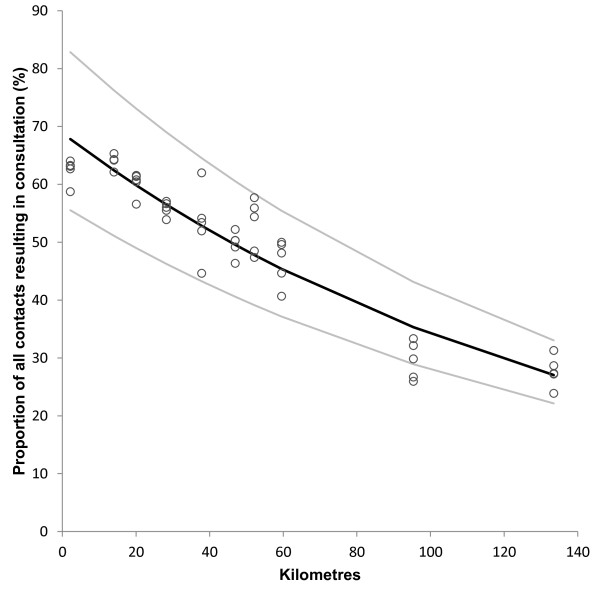
**Distance and proportion of contacts resulting in a face to face consultation at the casualty clinic.** Each point represents proportion of all contacts in one municipality for one year that resulted in a face-to-face doctor consultation. Regression curve (black line) for ln-transformed data with 95% confidence interval (grey lines). Ln (% contacts) = 4.32 –0.007*distance (km). N = 50, R^2^ = 0.89, p < 0.001.

Increasing distance was significantly correlated with decreasing frequency of all types of first actions taken (Table 4). The only exception was telephone consultations with doctor, which became slightly more frequent with increasing distance. The distance effect was most pronounced for consultations with a doctor. Figure 5 shows that the proportion of first contacts resulting in a face-to-face consultation significantly decreased with increasing distance from the casualty clinic. Only 28 per cent of contacts from Fyresdal resulted in consultation with doctor compared to 62 per cent in Arendal, OR 0.22 (95% CI 0.14 to 0.36).

**Table 4:** Distance and first actions taken

	**Change 0 to 50 km (%)**	**Constant**	**Coefficient (β)**	**95% CI for β**	**R**^**2**^	**p**
**Consultation doctor**	-55.0	5.54	-0.016	-0.017 to -0.015	0.96	<0.001
**Consultation others**	-47.8	0.43	-0.013	-0.120 to -0.001	0.13	0.020
	-16.5*	1.82*	-0.006*	-0.018 to -0.008*	0.36*	<0.001*
**Emergency ambulance with doctor**	-29.5	2.11	-0.007	-0.100 to -0.004	0.30	<0.001
**Doctor**’**s home visit**	-42.3	1.81	-0.011	-0.140 to -0.008	0.57	<0.001
	-24.4*	7.78*	-0.038*	-0.048 to -0.029*	0.58*	<0.001*
**Telephone consultation by doctor**	10.5	3.79	0.002	0.001 to 0.003	0.15	0.006
**Telephone by nurse**	-33.0	4.07	-0.008	-0.009 to -0.007	0.85	<0.001

Relative change in rates of different first actions taken after contact with the out-of-hours service when moving 50 kilometres away from the casualty clinic. Simple linear regression for the association between distance and the different priority grades performed (ln-transformed, except data marked *). N = 50.

Increasing distance was significantly correlated with decreasing total contact rate and decreasing consultation frequency for all age groups (see Additional file 2). The proportion of contacts for patients aged 66+ was increasing with increasing distance (see Additional file 3). The percentage of contacts with patients older than 80 years in the most distant municipality was significantly lower than expected from the other municipalities. When excluding this outlier, the rate ratios of all first contacts of this age group compared with younger patients significantly increased with increasing distance from the casualty clinic. Based on the linear regression equation (N = 45, R^2^ = 0.79, p < 0.001), rate ratios for all contacts among persons more than 80 years old went up from 1.3 to 2.5 when moving 50 kilometres away from the casualty clinic (0.025 per kilometre). In contrast, there was a slight decline (0.003 per kilometre) in rate ratios of contacts among patients aged 13 to 19 compared with all other ages (N = 50, R^2^ = -0.43, p < 0.001). Also the proportion of young adults aged 20 to 34 decreased with increasing distance (0.2% reduction per kilometre, N = 50, R^2^ = 0.22, p < 0.001). The percentages of the remaining age groups were not affected by distance to the casualty clinic (see Additional file 3).

Increasing distance was significantly correlated with decreasing proportion of females among patients contact contacting the out-of-hours service (0.045 percentage points reduction per kilometre, R^2^ = 0.27, p < 0.001).

The proportion of contacts that lacked at least one piece of information varied from 0.6 per cent in 2011 to 2.6 per cent in 2009. Analyses on main outcomes with non-weighted data resulted in minimal and non-significant changes (see Additional files 4 and 5).

## Discussion

Our findings confirm the hypothesis that increasing travel distance from population centroid of resident municipality to a casualty clinic is highly correlated with lower rates of face-to face consultations with a doctor, and with all other kinds of contacts with the casualty clinic. Only telephone consultations with a doctor increased slightly with increasing distance. The effect was most pronounced for non-urgent cases, but even acute events (red responses) were reduced by 22 per cent for people living 50 kilometres away. The proportion of first contacts that resulted in a face-to-face consultation with a doctor also decreased considerably with increasing distance. The trend was the same for all ages and both genders, but males and the oldest patients comprised a larger share of contacts with increasing distance. In contrast, teenagers living in the most remote municipalities had lower contact rates than expected. Multivariate analysis showed that availability of primary care doctors and education level were the only confounding factors that contributed to the variance in face-to-face consultation rate, although to a limited extent. We failed to identify any demographic or socioeconomic factors that influenced the rate of all first contacts with the out-of-hours service.

The data used in this study have many properties that make it well suited to study how geography affects the utilisation of out-of-hours services. The data set contains many cases and many relevant variables. It includes almost all activity year-round for five years in a large area with a wide range of distances. Additional information on potential demographic and socioeconomic confounding factors was obtained from reliable official sources.

However, great care should be taken when discussing causal relationships in studies like this, in order to avoid the Ecological fallacy [13]. Potential confounders were not analysed on individual level, but on data aggregated for year and municipality. There is a risk that important information might have disappeared in the aggregation process. It cannot be completely ruled out that some of our findings can be explained by unknown confounders. On the other hand, the strength of the associations observed, both quantitatively and by statistical significance, suggests a true causal relationship between distance and utilisation of out-of-hours services.

Another limitation is some inaccuracy of the Watchtower data. The information was most often recorded per telephone, and the patients were not identified in our database. Multiple contacts with the same patient on the same day were possible. The priority grade was based on initial triage performed by nurse upon initial contact [8], and did not necessarily reflect the actual need of all patients. However, we find it unlikely that these errors correlate with distance and thus cause recording bias.

The distance from the population centroid to the casualty clinic is a good proximity measure for all municipalities. A notable exception is Arendal city itself. Because the location of the clinic and the population centroid of Arendal municipality almost coincide, the estimated average travel distance is smaller than the actual one. There has been some discussion about the appropriateness of different distance measures. We performed post-hoc analyses on travel time and on distances from town halls in each municipality. No changes were observed when analysing alternative distance measures (see Additional files 6 and 7). Potential limiting traffic factors such as ferries, toll roads, mountain passes or extreme poor road quality are almost non-existent in Arendal out-of-hours district. The only two toll booths are both located at the outer borders, so that very few patients had to pass them to get to the casualty clinic.

The results of this study indicate that travel distance to the casualty clinic is a major barrier for the use of the out-of-hours service in this district. Both over-utilisation near the casualty clinic and under-utilisation in remote municipalities probably contribute to the large differences in contact and consultation rates. The vanishingly small impact of the investigated demographic and socioeconomic factors indicates that the travel costs in terms of time and money is the key factor when a patient decides whether to contact the out-of-hours service or not.

It should be of particular concern that also the rate of red responses and cases needing doctor assisted ambulance action also decreased with distance. We do not know how many of the most seriously ill patients that are taken directly to the hospital bypassing the casualty clinic, and whether this happens more often in rural areas, e.g. by ambulance helicopters. Rates of hip fractures and cardiovascular disease treated in hospital did not contribute to the variance of red response rate in a post-hoc multivariate analysis. The observed lower rate of the most acute cases in the remote municipalities raises serious safety questions. In a Scottish cohort study, the mortality risk from myocardial infarction increased twofold for patients living more than 14.5 kilometres away from hospital, non-admittance was a major contributing factor [14].

The proportion of the oldest patients increase with increasing distance, reflecting higher needs in this group, while the decrease in contact rate among teenagers may represent underutilisation of the out-of-hours service.

It would be interesting to know whether our results can predict the contact and consultation rate in primary emergency healthcare elsewhere. The effects on main outcomes are in the same magnitude as previous findings. In a study on the effects on distances to hospital in the UK, the inpatient acute emergency episode rate (unadjusted) was reduced by 42% for people living 40 kilometres away from the hospital [15]. In our model, moving 40 kilometres away from the casualty clinic reduce the consultation rate by 47%. A study from Northern Ireland showed that people living 34 kilometres away had an odds ratio of face-to-face consultation of 0.50 compared with patients living close to an out-of-hours primary care centre [4]. The authors of this study chose to present the association as linear, but like in our study, a negative exponential function was found to fit the data best.

The emergency primary health care in Norway is organised differently than in most other countries. General practitioners constitute the mainstay of the out-of-hours services. Alternatives to the publicly organised scheme are almost non-existent. Norway is also sparsely populated compared to most other European countries. Arendal casualty clinic has been specifically chosen to be a part of the Watchtower project, because it is a typical Norwegian inter-municipal out-of-hours service.

## Conclusion

This study has shown that increasing distance is associated with lower utilisation of out-of-hours services in a Norwegian district, even for the most acute cases. Our findings suggest that distance alone is more important for the variation in the use of casualty clinics than a number of relevant demographic and socio-economic factors. We believe that the results could be used to predict the activity of other similar districts, but to a less extent to out-of-hours services that are differently organised. Quasi-experimental studies of changes in organisation and distances to casualty clinics could support the results and would be welcome.

## Competing interests

The authors declare they have no competing interests.

## Authors’ contributions

GR contributed to the conception of the study, designed the study, performed statistical analyses and drafted the manuscript. EHH was responsible for the collection of data in the Watchtower project. SH contributed to the conception and design of the study. All authors participated in revising the manuscript and approving the final version.

## Pre-publication history

The pre-publication history for this paper can be accessed here:

http://www.biomedcentral.com/1472-6963/13/222/prepub

## Supplementary Material

Additional file 1 **Baseline data.** Demographic and selected socioeconomic baseline data for the municipalities served by Arendal out-of-hours district. Aggregated data for complete population. Click here for file

Additional file 2 **Contact rates in different age groups.** Bivariate correlation between rates of all contacts with casualty clinic (per 1 000 inhabitants per year) in different age groups and distance from population centroid. N = 50. Click here for file

Additional file 3 **Proportion of contacts by age.** Bivariate correlation between the proportion of contacts from each age group (%) and distance from population centroid (kilometres) N = 50. Click here for file

Additional file 4 **Analysis on non-weighted data, contact rates.** Simple linear regression. Weighted and non-weighted data, correction due to missing data (ref. text). Rate of all contact with casualty clinic by different distance from population centroid of municipalities to Arendal casualty clinic. N = 50. Click here for file

Additional file 5 **Analysis on non-weighted data, consultation rates.** Simple linear regression. On weighted and non-weighted data, correction due to missing data (ref. text). Rate of face to face consultations by different distance measures from municipalities to Arendal casualty clinic. N = 50. Click here for file

Additional file 6 **Alternative distance measures and contact rates.** Simple linear regression. Rate of all contacts by different distance measures from municipalities to Arendal casualty clinic. N = 50. Click here for file

Additional file 7 **Alternative distance measures and consultation rates.** Simple linear regression. Rate of face to face consultations by distance from population centroid of municipalities to Arendal casualty clinic. N = 50. Click here for file
