# Supplementary material for: Distance and utilisation of out-of-hours services in a Norwegian urban/rural district: an ecological study
Source: BMC Health Serv Res. 2013 Jun 17;13:222. doi: 10.1186/1472-6963-13-222 (PMC3703450; doi:10.1186/1472-6963-13-222)
Supplement: Additional file 1 — Baseline data. Demographic and selected socioeconomic baseline data for the municipalities served by Arendal out-of-hours district. Aggregated data for complete population. [file 1472-6963-13-222-S1.pdf]

	Name of municipality									
	Arendal	Froland	Grimstad	Tvedestrand	Vegårshei	Risør	Gjerstad	Åmli	Nissedal	Fyresdal
**Distance from population centroid to casualty clinic (kilometres)**	2.2	14.0	20.1	28.2	37.8	46.9	52.1	59.5	95.4	133.5
**Population 2011**	42 229	5 127	20 823	5 969	1 922	6 871	2 497	1 829	1 405	1 351
**Primary care doctors per 10 000 inhabitants (Mean 2007-2011)**	5.6	9.0	9.3	11.0	10.2	7.3	9.8	18.1	24.3	14.1
**Population >80 years (2011. %)**	4.5	3.5	3.5	5.5	5.9	6.0	5.4	5.5	6.1	6.2
**Primary school as highest finished education level (%)**	28.4	33.8	26.7	29.6	28.6	30.5	39.3	34.8	30.8	27.8
**Median gross income 2010 (1 000 NOK)**	298	288	293	275	281	268	264	264	275	277
**Income inequality (Gini coefficient) 2009**	0.22	0.21	0.27	0.21	0.20	0.22	0.19	0.21	0.20	0.19
