# Supplementary material for: Distance and utilisation of out-of-hours services in a Norwegian urban/rural district: an ecological study
Source: BMC Health Serv Res. 2013 Jun 17;13:222. doi: 10.1186/1472-6963-13-222 (PMC3703450; doi:10.1186/1472-6963-13-222)
Supplement: Additional file 3 — Proportion of contacts by age. Bivariate correlation between the proportion of contacts from each age group (%) and distance from population centroid (kilometres) N = 50. [file 1472-6963-13-222-S3.pdf]

Age group	0-5 years	6-12 years	13-19 years	20 - 34 years	35-49 years	Rate 50-66 years	67-79 years	80 years
Pearson r	0.051	0.131	-0.654	-0.475	0.231	0.105	0.652	0.510
p	0.728	0.363	<0.001	<0.001	0.107	0.470	<0.001	<0.001
