# Supplementary material for: Distance and utilisation of out-of-hours services in a Norwegian urban/rural district: an ecological study
Source: BMC Health Serv Res. 2013 Jun 17;13:222. doi: 10.1186/1472-6963-13-222 (PMC3703450; doi:10.1186/1472-6963-13-222)
Supplement: Additional file 5 — Analysis on non-weighted data, consultation rates. Simple linear regression. On weighted and non-weighted data, correction due to missing data (ref. text). Rate of face to face consultations by different distance measures from municipalities to Arendal casualty clinic. N = 50. [file 1472-6963-13-222-S5.pdf]

	Constant	β	95% CI for β	R^2	Pearson r
**Weighted data**	5.54	-0.016	-0.017 to -0.015	0.96	-0.98
**Distance from population centroid (kilometres)**					
(Main outcome)					
**Non-weighted data**	5.54	-0.016	-0.017 to -0.015	0.96	-0.98
**Distance from population centroid (kilometres)**					
