# Supplementary material for: Distance and utilisation of out-of-hours services in a Norwegian urban/rural district: an ecological study
Source: BMC Health Serv Res. 2013 Jun 17;13:222. doi: 10.1186/1472-6963-13-222 (PMC3703450; doi:10.1186/1472-6963-13-222)
Supplement: Additional file 6 — Alternative distance measures and contact rates. Simple linear regression. Rate of all contacts by different distance measures from municipalities to Arendal casualty clinic. N = 50. [file 1472-6963-13-222-S6.pdf]

	Constant	β	95% CI for β	R^2	Pearson r
**Distance from population centroid (kilometres)** (Main outcome)	5.92	-0.009	-0.010 to -0.009	0.95	-0.98
**Driving time from population centroid (minutes)**	5.79	-0.008	-0.009 to -0.007	0.82	-0.91
**Distance from Town hall (kilometres)**	5.91	-0.009	-0.010 to 0.009	0.94	0.97
