# Supplementary material for: Distance and utilisation of out-of-hours services in a Norwegian urban/rural district: an ecological study
Source: BMC Health Serv Res. 2013 Jun 17;13:222. doi: 10.1186/1472-6963-13-222 (PMC3703450; doi:10.1186/1472-6963-13-222)
Supplement: Additional file 7 — Alternative distance measures and consultation rates. Simple linear regression. Rate of face to face consultations by distance from population centroid of municipalities to Arendal casualty clinic. N = 50. [file 1472-6963-13-222-S7.pdf]

	Constant	β	95% CI for β	R^2	Pearson r
**Distance from population centroid (kilometres)**<br><br>(Main outcome)	5.54	-0.016	-0.017 to -0.015	0.96	-0.98
**Driving time from population centroid (minutes)**	5.32	-0.014	-0.016 to -0.012	0.82	-0.91
**Distance from Town hall (kilometres)**	5.53	-0.016	-0.016 to 0.017	0.94	0.97
